# Supplementary material for: Polypharmacy and associated factors in South Korean elderly patients with dementia: An analysis using National Health Insurance claims data
Source: PLoS One. 2024 Apr 25;19(4):e0302300. doi: 10.1371/journal.pone.0302300 (PMC11045087; doi:10.1371/journal.pone.0302300)
Supplement: S1 Table — (DOCX) [file pone.0302300.s001.docx]

**Supplimentary Tables**

**S1 Table. Codes used to define patients with dementia**

| **Code** | **Description** |
| --- | --- |
| **KCD code**^1)^ |  |
| F00 | “Dementia in Alzheimer’s disease” |
| F01 | “Vascular dementia” |
| F02 | “Dementia in other diseases classified elsewhere” |
| F03 | “Unspecified dementia” |
| G30 | “Alzheimer’s disease” |
| G31.82 | “Dementia with Lewy bodies” |
| **ATC code**^2)^ |  |
| N06DA02 | Donepezil |
| N06DA03 | Rivastigmine |
| N06DA04 | Galantamine |
| N06DX01 | Memantine |

KCD: Korean Standard Classification of Diseases and Causes of Death, ATC: Anatomical Therapeutic Chemical

Sources: 1) Statistics Korea. Korean Standard Classification of Diseases [Internet]. Daejeon: Statistics Korea; 2023. Available from: <http://kssc.kostat.go.kr/ksscNew_web/ekssc/main/main.do>

2) WHO Collaborating Centre for Drug Statistics Methodology. ATC/DDD Index. WHO Collaborating Centre for Drug Statistics Methodology; 2023. Available from: <https://www.whocc.no/atc_ddd_index>
