# Supplementary material for: Polypharmacy and associated factors in South Korean elderly patients with dementia: An analysis using National Health Insurance claims data
Source: PLoS One. 2024 Apr 25;19(4):e0302300. doi: 10.1371/journal.pone.0302300 (PMC11045087; doi:10.1371/journal.pone.0302300)
Supplement: S3 Table — (DOCX) [file pone.0302300.s003.docx]

**S3 Table. Distribution of comorbidities among patients with dementia based on polypharmacy status**

| **Characteristics** | Dementia | | **Polypharmacy (5+)** | | | | **Excessive polypharmacy (10+)** | | | |
| --- | --- | --- | --- | --- | --- | --- | --- | --- | --- | --- |
|  | **n** | **%** | | **n** | **%** | ***P*-value^*^** | | **n** | **%** | ***P*-value^*^** |
| **Total** | 57,346 | (100.0) | | 40,318 | (100.0) |  | | 13,581 | (100.0) |  |
| **CCI disease** |  |  | |  |  |  | |  |  |  |
| **Congestive heart failure** | 9,437 | (16.5) | | 7,836 | (19.4) | <0.0001 | | 3,807 | (28.0) | <0.0001 |
| **Dementia** | 57,222 | (99.8) | | 40,235 | (99.8) | 0.4108 | | 13,557 | (99.8) | 0.2565 |
| **Chronic pulmonary disease** | 20,983 | (36.6) | | 16,085 | (39.9) | <0.0001 | | 6,757 | (49.8) | <0.0001 |
| **Rheumatologic disease** | 2,698 | (4.7) | | 2,238 | (5.6) | <0.0001 | | 1,074 | (7.9) | <0.0001 |
| **Mild liver disease** | 16,782 | (29.3) | | 12,786 | (31.7) | <0.0001 | | 5,133 | (37.8) | <0.0001 |
| **Diabetes with chronic complications** | 9,022 | (15.7) | | 7,832 | (19.4) | <0.0001 | | 4,014 | (29.6) | <0.0001 |
| **Hemiplegia or paraplegia** | 2,871 | (5.0) | | 2,087 | (5.2) | 0.0041 | | 756 | (5.6) | 0.0006 |
| **Renal disease** | 3,990 | (7.0) | | 3,160 | (7.8) | <0.0001 | | 1,611 | (11.9) | <0.0001 |
| **Any malignancy, including leukemia and lymphoma, except malignant neoplasm of skin** | 3,646 | (6.4) | | 2,590 | (6.4) | 0.3187 | | 993 | (7.3) | 0.0001 |
| **Moderate or severe liver disease** | 282 | (0.5) | | 208 | (0.5) | 0.2034 | | 82 | (0.6) | 0.0326 |
| **Metastatic solid tumor** | 374 | (0.7) | | 244 | (0.6) | 0.0315 | | 75 | (0.6) | 0.0977 |
| **AIDS/HIV** | - | - | | - | - |  | | - | - |  |
| **Other comorbidities related with dementia** |  |  | |  |  |  | |  |  |  |
| **Hypertension** | 41,992 | (73.2) | | 32,601 | (80.9) | <0.0001 | | 12,071 | (88.9) | <0.0001 |
| **Depression** | 24,707 | (43.1) | | 18,878 | (46.8) | <0.0001 | | 7,478 | (55.1) | <0.0001 |
| **Mental health related disease except dementia** | 43,982 | (76.7) | | 32,430 | (80.4) | <0.0001 | | 11,799 | (86.9) | <0.0001 |

^*^Chi-square test of differences between patients with and without polypharmacy (5+)

^**^Chi-square test of differences between patients with and without excessive polypharmacy (10+)

CCI: Charlson comorbidity index, AIDS: acquired immunodeficiency syndrome, HIV: human immunodeficiency virus

Note: 1. The definition of dementia comorbidity under CCI disease category in this table was based on the definition of dementia provided by the CCI algorithm.

2. Except for excluded diseases, all diseases documented in the 2019 medical record were considered for calculating the CCI. Consequently, dementia comorbidities were identified in some non-dementia patients, specifically cases where dementia was present in a disease code other than the primary or secondary disease code.

3. The disease codes that define other comorbidities related with dementia were as follows. Hypertension: I10 – I15, depression: F32, F33, mental health related disease except dementia: codes starting with F, except F00, F01, F02, F03
