# Supplementary material for: Polypharmacy and associated factors in South Korean elderly patients with dementia: An analysis using National Health Insurance claims data
Source: PLoS One. 2024 Apr 25;19(4):e0302300. doi: 10.1371/journal.pone.0302300 (PMC11045087; doi:10.1371/journal.pone.0302300)
Supplement: S4 Table — (DOCX) [file pone.0302300.s004.docx]

**S4 Table. Characteristics of patients without dementia based on polypharmacy status**

| **Characteristics** | | **Non-Dementia** | | **Polypharmacy (5+)** | | | **Excessive polypharmacy (10+)** | | | |
| --- | --- | --- | --- | --- | --- | --- | --- | --- | --- | --- |
|  |  | **n** | **%** | **n** | **%** | ***P*-value^*^** | **n** | **%** | ***P*-value^**^** |  |
| **Total** | | 810,331 | (100.0) | 303,149 | (100.0) |  | 54,977 | (100.0) |  |  |
| **Sex** | **Male** | 370,445 | (45.7) | 137,109 | (45.2) | <0.0001 | 25,329 | (46.1) | 0.0820 |  |
|  | **Female** | 439,886 | (54.3) | 166,040 | (54.8) |  | 29,648 | (53.9) |  |  |
| **Age** | **60–64** | 272,114 | (33.6) | 64,585 | (21.3) | <0.0001 | 7,837 | (14.3) | <0.0001 |  |
|  | **65–69** | 186,263 | (23.0) | 63,826 | (21.1) |  | 9,610 | (17.5) |  |  |
|  | **70–74** | 141,559 | (17.5) | 61,378 | (20.2) |  | 11,635 | (21.2) |  |  |
|  | **75–79** | 110,631 | (13.7) | 57,317 | (18.9) |  | 12,716 | (23.1) |  |  |
|  | **80–84** | 67,628 | (8.3) | 38,219 | (12.6) |  | 9,106 | (16.6) |  |  |
|  | **85–89** | 27,411 | (3.4) | 15,338 | (5.1) |  | 3,576 | (6.5) |  |  |
|  | **90–91** | 4,725 | (0.6) | 2,486 | (0.8) |  | 497 | (0.9) |  |  |
| **Disability** | **Severe** | 25,573 | (3.2) | 12,515 | (4.1) | <0.0001 | 3,374 | (6.1) | <0.0001 |  |
|  | **Mild** | 77,180 | (9.5) | 40,461 | (13.3) |  | 10,235 | (18.6) |  |  |
|  | **None** | 707,578 | (87.3) | 250,173 | (82.5) |  | 41,368 | (75.2) |  |  |
| **Region** | **Seoul-Metro** | 363,581 | (44.9) | 126,701 | (41.8) | 0.0180 | 20,227 | (36.8) | <0.0001 |  |
|  | **Chungcheong** | 88,404 | (10.9) | 34,626 | (11.4) |  | 6,459 | (11.7) |  |  |
|  | **Honam** | 92,171 | (11.4) | 41,460 | (13.7) |  | 9,480 | (17.2) |  |  |
|  | **Gyeongsang** | 226,108 | (27.9) | 84,871 | (28.0) |  | 16,149 | (29.4) |  |  |
|  | **Gangwon-Jeju** | 40,067 | (4.9) | 15,491 | (5.1) |  | 2,662 | (4.8) |  |  |
| **Income level** | **Medical Aid** | 49,558 | (6.1) | 28,045 | (9.3) | <0.0001 | 9,000 | (16.4) | <0.0001 |  |
|  | **NHI 1st** | 132,485 | (16.3) | 46,703 | (15.4) |  | 7,987 | (14.5) |  |  |
|  | **NHI 2nd** | 93,088 | (11.5) | 31,178 | (10.3) |  | 5,035 | (9.2) |  |  |
|  | **NHI 3rd** | 120,417 | (14.9) | 42,275 | (13.9) |  | 6,997 | (12.7) |  |  |
|  | **NHI 4th** | 164,208 | (20.3) | 59,353 | (19.6) |  | 9,578 | (17.4) |  |  |
|  | **NHI 5th (richest)** | 250,575 | (30.9) | 95,595 | (31.5) |  | 16,380 | (29.8) |  |  |
| **Type of long-term care benefits** | **Institutional care** | 2,242 | (0.3) | 1,385 | (0.5) | <0.0001 | 351 | (0.6) | <0.0001 |  |
|  | **Home care** | 17,680 | (2.2) | 12,257 | (4.0) |  | 4,352 | (7.9) |  |  |
|  | **None** | 790,409 | (97.5) | 289,507 | (95.5) |  | 50,274 | (91.4) |  |  |
| **CCI** | **≤2** | 632,772 | (78.1) | 191,894 | (63.3) | <0.0001 | 24,769 | (45.1) | <0.0001 |  |
|  | **3–4** | 131,726 | (16.3) | 79,212 | (26.1) |  | 18,851 | (34.3) |  |  |
|  | **≥5** | 45,833 | (5.7) | 32,043 | (10.6) |  | 11,357 | (20.7) |  |  |

^*^Chi-square test of differences between patients with and without polypharmacy (5+)

^**^Chi-square test of differences between patients with and without excessive polypharmacy (10+)

NHI: National Health Insurance, CCI: Charlson comorbidity index
