# Supplementary material for: Polypharmacy and associated factors in South Korean elderly patients with dementia: An analysis using National Health Insurance claims data
Source: PLoS One. 2024 Apr 25;19(4):e0302300. doi: 10.1371/journal.pone.0302300 (PMC11045087; doi:10.1371/journal.pone.0302300)
Supplement: S6 Table — (DOCX) [file pone.0302300.s006.docx]

**S6 Table. Factors associated with polypharmacy: comparison between patients with and without dementia**

| **Variable** | | **Non-dementia patients**  **(n=810,331)** | | | | | | **Dementia patients**  **(n=57,346)** | | | | | |
| --- | --- | --- | --- | --- | --- | --- | --- | --- | --- | --- | --- | --- | --- |
|  |  | **Polypharmacy (5+)** | | | **Excessive polypharmacy (10+)** | | | **Polypharmacy (5+)** | | | **Excessive polypharmacy (10+)** | | |
|  |  | **OR** | **95% CI** | ***P*-value** | **OR** | **95% CI** | ***P*-value** | **OR** | **95% CI** | ***P*-value** | **OR** | **95% CI** | ***P*-value** |
| **Sex** | **Male (ref.)** |  |  |  |  |  |  |  |  |  |  |  |  |
|  | **Female** | 0.984 | 0.974–0.993 | <0.0001 | 0.926 | 0.909-0.943 | <0.0001 | 1.051 | 1.019–1.105 | 0.0038 | 0.945 | 0.905–0.987 | 0.0114 |
| **Age** | **60–64 (ref.)** |  |  |  |  |  |  |  |  |  |  |  |  |
|  | **65–69** | 1.598 | 1.576-1.619 | <0.0001 | 1.647 | 1.596-1.699 | <0.0001 | 1.195 | 1.066–1.339 | 0.0022 | 1.187 | 1.031–1.365 | 0.0169 |
|  | **70–74** | 2.251 | 2.219-2.284 | <0.0001 | 2.490 | 2.416-2.567 | <0.0001 | 1.482 | 1.334–1.646 | <0.0001 | 1.545 | 1.360–1.756 | <0.0001 |
|  | **75–79** | 3.054 | 3.006-3.102 | <0.0001 | 3.364 | 3.263-3.468 | <0.0001 | 1.725 | 1.563–1.905 | <0.0001 | 1.698 | 1.504–1.918 | <0.0001 |
|  | **80–84** | 3.543 | 3.477-3.610 | <0.0001 | 3.735 | 3.612-3.862 | <0.0001 | 1.583 | 1.435–1.746 | <0.0001 | 1.523 | 1.349–1.719 | <0.0001 |
|  | **85–89** | 3.251 | 3.163-3.341 | <0.0001 | 3.259 | 3.115-3.410 | <0.0001 | 1.247 | 1.127–1.379 | <0.0001 | 1.184 | 1.045–1.341 | 0.0082 |
|  | **90–91** | 2.685 | 2.524-2.856 | <0.0001 | 2.330 | 2.103-2.581 | <0.0001 | 1.027 | 0.910–1.160 | 0.6647 | 0.946 | 0.811–1.102 | 0.4756 |
| **Disability** | **Severe** | 1.125 | 1.094-1.157 | <0.0001 | 1.311 | 1.257-1.367 | <0.0001 | 0.867 | 0.812–0.926 | <0.0001 | 0.968 | 0.902–1.039 | 0.3713 |
|  | **Mild** | 1.502 | 1.478-1.527 | <0.0001 | 1.656 | 1.615-1.698 | <0.0001 | 1.248 | 1.187–1.312 | <0.0001 | 1.318 | 1.254–1.386 | <0.0001 |
|  | **None (ref)** |  |  |  |  |  |  |  |  |  |  |  |  |
| **Region** | **Seoul-Metro (ref)** |  |  |  |  |  |  |  |  |  |  |  |  |
|  | **Chungcheong** | 1.163 | 1.144-1.182 | <0.0001 | 1.275 | 1.237-1.315 | <0.0001 | 0.908 | 0.856–0.964 | 0.0015 | 0.965 | 0.904–1.030 | 0.2864 |
|  | **Honam** | 1.388 | 1.366-1.410 | <0.0001 | 1.688 | 1.643-1.735 | <0.0001 | 0.888 | 0.841–0.938 | <0.0001 | 1.151 | 1.086–1.219 | <0.0001 |
|  | **Gyeongsang** | 1.087 | 1.075-1.100 | <0.0001 | 1.252 | 1.224-1.280 | <0.0001 | 0.936 | 0.894–0.980 | 0.0051 | 1.036 | 0.984–1.089 | 0.1757 |
|  | **Gangwon-Jeju** | 1.069 | 1.045-1.094 | <0.0001 | 1.040 | 0.996-1.087 | 0.0768 | 0.912 | 0.837–0.994 | 0.0359 | 0.928 | 0.843–1.021 | 0.1256 |
| **Income level** | **Medical Aid** | 1.894 | 1.854-1.934 | <0.0001 | 2.671 | 2.592-2.753 | <0.0001 | 1.210 | 1.139–1.286 | <0.0001 | 1.695 | 1.586–1.799 | <0.0001 |
|  | **NHI 1st** | 1.071 | 1.055-1.087 | <0.0001 | 1.147 | 1.115-1.181 | <0.0001 | 0.932 | 0.880–0.986 | 0.0141 | 1.077 | 1.011–1.147 | 0.0209 |
|  | **NHI 2nd** | 1.059 | 1.042-1.078 | <0.0001 | 1.116 | 1.079-1.155 | <0.0001 | 0.936 | 0.832–0.961 | 0.0024 | 1.009 | 0.930–1.096 | 0.8224 |
|  | **NHI 3rd** | 1.076 | 1.060-1.093 | <0.0001 | 1.108 | 1.075-1.142 | <0.0001 | 0.912 | 0.892–1.010 | 0.1020 | 1.013 | 0.945–1.087 | 0.7103 |
|  | **NHI 4th** | 1.071 | 1.058-1.087 | <0.0001 | 1.054 | 1.025-1.083 | 0.0002 | 1.073 | 0.943–1.053 | 0.8927 | 1.031 | 0.970–1.097 | 0.3253 |
|  | **NHI 5th (richest, ref.)** |  |  |  |  |  |  |  |  |  |  |  |  |
| **Type of long-term care benefits** | **Institutional care** | 1.237 | 1.129-1.355 | <0.0001 | 1.081 | 0.956-1.222 | 0.2160 | 1.459 | 1.376–1.547 | <0.0001 | 1.049 | 0.986–1.117 | 0.1325 |
|  | **Home care** | 1.716 | 1.656-1.778 | <0.0001 | 1.895 | 1.819-1.974 | <0.0001 | 1.304 | 1.249–1.362 | <0.0001 | 1.226 | 1.170–1.283 | <0.0001 |
|  | **None (ref.)** |  |  |  |  |  |  |  |  |  |  |  |  |
| **CCI** | **≤2 (ref.)** |  |  |  |  |  |  |  |  |  |  |  |  |
|  | **3–4** | 3.241 | 3.200-3.283 | <0.0001 | 3.549 | 3.477-3.623 | <0.0001 | 1.941 | 1.860–2.026 | <0.0001 | 2.454 | 2.320–2.595 | <0.0001 |
|  | **≥5** | 4.313 | 4.221-4.406 | <0.0001 | 5.936 | 5.785-6.092 | <0.0001 | 2.861 | 2.729–3.000 | <0.0001 | 4.450 | 4.211–4.704 | <0.0001 |

Ref.: reference

NHI: National Health Insurance, CCI: Charlson comorbidity index
