# Supplementary material for: Polypharmacy and associated factors in South Korean elderly patients with dementia: An analysis using National Health Insurance claims data
Source: PLoS One. 2024 Apr 25;19(4):e0302300. doi: 10.1371/journal.pone.0302300 (PMC11045087; doi:10.1371/journal.pone.0302300)
Supplement: S7 Table — (DOCX) [file pone.0302300.s007.docx]

**S7 Table. Distribution of medications included in polypharmacy**

|  | Dementia | | | | Non-dementia | | | |  |
| --- | --- | --- | --- | --- | --- | --- | --- | --- | --- |
| **ATC code** | Polypharmacy (5+) | | Excessive polypharmacy (10+) | | Polypharmacy (5+) | | Excessive polypharmacy (10+) | |  |
|  | **n** | **%** | **n** | **%** | **n** | **%** | **n** | **%** | |
| **Total** | 40,318 | (100.0) | 13,581 | (100.0) | 303,149 | 100.0 | 54,977 | 100.0 | |
| **N06D (Anti-dementia drugs)** | 32,653 | (81.0) | 11,534 | (84.9) | 36,791 | (12.1) | 9,689 | (17.6) | |
| **N06DA (Anticholinesterases)** | 29,352 | (72.8) | 10,405 | (76.6) | - |  | - | - | |
| **N06DX (Other anti-dementia drugs)** | 11,164 | (27.7) | 4,202 | (30.9) | 36,791 | (12.1) | 9,689 | (17.6) | |
| **N05A (Antipsychotics)** | 9,040 | (22.4) | 3,500 | (25.8) | 5,098 | (1.7) | 1,948 | (3.5) | |
| **N05B (Anxiolytics)** | 16,542 | (41.0) | 6,978 | (51.4) | 116,914 | (38.6) | 28,473 | (51.8) | |
| **N05C (Hypnotics and sedatives)** | 6,197 | (15.4) | 2,840 | (20.9) | 36,424 | (12.0) | 10,419 | (19.0) | |
| **N03A (Antiepileptics)** | 11,435 | (28.4) | 5,516 | (40.6) | 63,615 | (21.0) | 19,872 | (36.1) | |
| **N06A (Antidepressants)** | 16,154 | (40.1) | 6,934 | (51.1) | 54,358 | (17.9) | 17,168 | (31.2) | |
| **N02 (Analgesics)** | 24,200 | (60.0) | 9,378 | (69.1) | 213,076 | (70.3) | 43,516 | (79.2) | |

Note: This table was based on whether each medication is present among the mediation combinations on days when patients with polypharmacy take 5 or more medications simultaneously, and among the medication combinations on days when patients with excessive polypharmacy take 10 or more medications simultaneously
