# Supplementary material for: Polypharmacy and associated factors in South Korean elderly patients with dementia: An analysis using National Health Insurance claims data
Source: PLoS One. 2024 Apr 25;19(4):e0302300. doi: 10.1371/journal.pone.0302300 (PMC11045087; doi:10.1371/journal.pone.0302300)
Supplement: Attachment — Submitted filename: Response to Reviewers.docx [file pone.0302300.s008.docx]

Dear Editor,

Thank you for providing us with the opportunity to resubmit our manuscript titled “Polypharmacy and associated factors in South Korean elderly patients with dementia: An analysis using National Health Insurance claims data.”

We also thank the reviewers for their efforts and useful comments, which greatly improved the completeness and quality of the paper.

**Response to Comments from the Editor**

**Comment 1.**

**The use of the term “polypharmacy patterns” is not tally with the results and discussion. No results were reported about the types of medications or dosing regimens. Therefore, please remove the word patterns and stick to “polypharmacy” throughout the manuscript.**

Thank you for your reasonable comment. As suggested, we revised the text to avoid the expression “polypharmacy patterns” and used the expression “polypharmacy” throughout the text. The title was also revised to delete the word “patterns.”

Revised title:

“Polypharmacy and associated factors in South Korean elderly patients with dementia: An analysis using National Health Insurance claims data”

Revised text on page 2, line 22 – 23:

“This study aimed to characterize polypharmacy and associated factors among elderly patients with dementia in South Korea…”

Revised text on page 2, line 29 – 30:

“We compared the prevalence of polypharmacy between patients with and without dementia and identified the associated factors using a logistic regression model.”

Revised text on page 4 – 5, line 67 – 69:

“Previous research has primarily focused on nursing home settings or potentially inappropriate medications, while studies on polypharmacy and associated factors in outpatient patients with dementia are relatively scarce.”

Revised text on page 4, line 78 – 81:

“The study of polypharmacy in patients with dementia could provide valuable information not only for clinical practitioners and policy makers in Korea, where the population is aging rapidly, but also in other countries with slower population aging rates.”

Revised text on page 4, line 85 – 88:

“To bridging this gap in knowledge, we aimed to determine the status and associated factors of polypharmacy among outpatients with dementia in 2019, using data sourced from the National Health Insurance Service (NHIS)-Senior cohort database.”

Revised text on page 16, line 268 – 271:

“This study examined the polypharmacy status of patients with dementia who were prescribed oral medications during outpatient treatment in 2019 and identified the associated factors using the NHIS-Senior cohort database, comprising representative data from a public single-payer in South Korea.”

**Comment 2.**

**L150: to stay consistent, please report the percentages for polypharmacy instead of non-polypharmacy.**

The sentence was revised as follows, with additional information provided for clarity.

Revised text on page 10, line 207 – 209:

“Polypharmacy was recorded in 70.3% and 37.4% of patients with and without dementia, respectively, whereas excessive polypharmacy was recorded in 23.7% and 6.8% of patients with and without dementia, respectively (Table 1).”

**Comment 3.**

**Do not repeat the results in the text and tables. Just highlight the important findings in the text or findings not stated in the tables.**

We agree that the original text may have repeated some results from the tables, and have revised the descriptions of the tables to focus on important findings not stated in the tables.

Revised text on page 10, line 199 – 209:

“The patients with and without dementia showed significant differences in several characteristics (Table 1). Specifically, the dementia group was, on average, 10 years older than the non-dementia group. Additionally, 10.0% of the dementia group fell into the 60s age range, compared to 56.6% of the non-dementia group. The proportion of individuals with disabilities in the dementia group was 27.8%, which was more than twice that in the non-dementia group (12.7%). Long-term care benefits were provided to 48.3% of patients with dementia, but only 2.5% of patients without dementia. Nearly 33.0% and 78.1% of patients with and without dementia had a CCI score ≤2, respectively. (The detailed distribution of comorbidities included in the CCI is presented in S2 Table.) Polypharmacy was recorded in 70.3% and 37.4% of patients with and without dementia, respectively, whereas excessive polypharmacy was recorded in 23.7% and 6.8% of patients with and without dementia, respectively (Table 1).”

Revised text on page 11, line 214 – 225:

“There was no significant difference in the distribution of sexes between patients with and without polypharmacy among dementia group (Table 2). However, the proportion of males was significantly higher in patients with excessive polypharmacy than in those without excessive polypharmacy (34.0% compared to 31.4%). Mild disability was observed among 23.0% of patients with excessive polypharmacy, which was significantly higher than that among patients without excessive polypharmacy (17.3%). Medical Aid beneficiaries were 20.8% of patients with excessive polypharmacy (compared to 12.9% of patients without excessive polypharmacy) and 15.8% of patients with polypharmacy (compared to 12.2% of patients without polypharmacy). In terms of long-term care benefits, home care benefits were provided to 36.5% and 32.2% of patients with and without excessive polypharmacy, respectively. Patients with polypharmacy and excessive polypharmacy showed a high CCI. A CCI score≥5 was observed in 48.5% and 25.8% of patients with and without excessive polypharmacy, respectively (Table 2).”

Revised text on page 14, line 238 – 249:

“The regression analysis showed that the likelihood of experiencing polypharmacy in patients with dementia increased significantly with age up to 70 (p<0.05), whereas it decreased at ages above 80. Those with mild disability were significantly more likely to experience polypharmacy than those without disabilities. Conversely, patients with severe disability had a significantly lower likelihood of experiencing polypharmacy. Compared to patients in the fifth NHI income quintile, Medical Aid beneficiaries with dementia were significantly more likely to experience polypharmacy [odds ratio (OR), 1.206; 95% confidence interval (CI), 1.135–1.281) and excessive polypharmacy (OR, 1.688; 95% CI, 1.590–1.792). Compared to patients who did not receive long-term care benefits, those who received home care had a significantly higher likelihood of polypharmacy and excessive polypharmacy, while those who received institutional care only had a significantly higher likelihood of experiencing polypharmacy. We found that the likelihood of experiencing polypharmacy and excessive polypharmacy increased with dementia duration and CCI score (Table 3).”

**Comment 4.**

**Table 1: Add a comparison of the mean age between the two groups.**

We added the mean age to Table 1, and also added a description in the text. We considered adding the mean age to Table 2 but, because the difference in mean age was not substantial, we avoided this revision to keep the table from exceeding one page. For reference, the average age (standard deviation) of each group was 79.6 years (7.0) in the dementia group, 79.6 years (6.8) in dementia patients with polypharmacy, and 79.3 years (6.5) in dementia patients with excessive polypharmacy.

**Comment 5.**

**Include the number of patients in the table captions.**

We added the number of patients to the caption of Table 3. We did not add it to Tables 1 and 2 because the number of patients in each group is included inside the table.

**Comment 6.**

**Make the abstract structured with conclusion.**

We structured the abstract by adding subheadings and separated the conclusion.

Revised Abstract on page 2 – 3, line 17 – 41:

The revised abstract is not cited here.

**Comment 7.**

**English language proofreading is needed.**

The manuscript was revised by an English language professional. We attached the certificate of English language proofreading.

**Response to the Comments from Reviewer 1**

**Major comments:**

**Comment 1.**

**The goal announced is “to discern patterns and associated factors of polypharmacy among outpatient suffering from dementia”. Results and discussion sections show first a comparison between dementia vs non-dementia patients then polypharmacy vs non-polypharmacy in patients suffering from dementia. Both groups can be compared to achieve the announced goal, but they answer different questions. Thus, the author should either improve the formulation of the goal/question and reorganize the methods, results, and discussion sections accordingly.**

Thank you for your important advice. The main objective of this study was to elucidate the characteristics of polypharmacy in patients with dementia. Accordingly, we compared the characteristics and prevalence of polypharmacy between patients with and without dementia to identify key factors, such as age and comorbidities. The purpose of the study was revised to clearly highlight this aspect, and the Methods, Results, and Discussion sections were supplemented for clarity.

Revised text on page 5, line 85 – 89:

“To bridging this gap in knowledge, we aimed to determine the status and associated factors of polypharmacy among outpatients with dementia in 2019, using data sourced from the National Health Insurance Service (NHIS)-Senior cohort database. To elucidate the characteristics, prevalence, and risk factors of polypharmacy in dementia, we compared the data between patients with and without dementia.”

Revised text on page 8, line 171 – 174:

“For comparison, the second and third analyses were also conducted in patients without dementia; however, considering the difficulty in defining the duration of dementia in these patients, the analyses excluded this variable, and again, for comparison, the third analysis was also conducted excluding this variable in patients with dementia.”

Revised text on page 14, line 233 – 235:

“In patients without dementia, we observed higher mild disability, Medical Aid, home care, and CCI scores in patients with polypharmacy and excessive polypharmacy than those without, consistent with the findings for patients with dementia (S4 Table).”

Revised text on page 15, line 253 – 259:

“Excluding the duration of dementia, the direction of influence of age, disability, Medical Aid beneficiaries, and CCI on polypharmacy and excessive polypharmacy and excessive polypharmacy was similar between patients with and without dementia, but the magnitude of the influence was greater in patients without dementia. In contrast, patients without dementia but with severe disabilities were significantly more likely to have polypharmacy and excessive polypharmacy than those without disabilities. The likelihood of excessive polypharmacy was significantly higher in the lower NHI income groups among patients without dementia (S6 Table).”

Revised text on page 18, line 332 – 344:

“Finally, our comparison of patients with and without dementia elucidated the characteristics of polypharmacy in this cohort along with important factors that affect its prevalence in patients with dementia. Since age, disability, Medical Aid beneficiaries, and CCI had greater effects on polypharmacy in patients without dementia (S6 Table), dementia itself may be a risk factor for polypharmacy. The higher risk for polypharmacy in patients with dementia compared to those without is consistent with findings from the United Kingdom (patients aged ≥65 years registered at general practices) [8] and United States (outpatients aged ≥65 years) [21], though the cohorts in these studies had different demographic profiles and healthcare systems. Additionally, the different directions with which patient characteristics affected polypharmacy, such as excessive polypharmacy increasing with decreasing income quintiles and the presence of severe disabilities among patients without dementia, suggests that some factors may have qualitatively different effects in patients with and without dementia. This in turn suggests that differentiated approaches may be required to treat these two populations.”

**Comment 2.**

**In line with the previous comment, the limit discussed l. 274 might be the most significant. Yet, it is discussed as the last one and the justification provided is not satisfactory. It would be possible either to attribute the value 0 for “Duration of dementia” to negate the variable for non-demented patients or to treat missing values for this variable as a level (if there are no missing values in the dementia patients’ group).**

Thank you for this important advice. We added the results for the logistic regression analysis on the factors influencing polypharmacy in patients without dementia as supplementary material. Since the duration of dementia was ambiguous in these patients, the analysis was conducted without the duration of dementia; for comparison, we also presented the analysis results without this variable for patients with dementia. However, the duration variable is important in the dementia group. For this reason, the results for the non-dementia group, were not included in Table 3 but presented in a supplementary table. For reference, we also considered conducting the logistic regression analysis with the dementia and non-dementia groups combined, treating dementia as a binary variable. However, some variables had different directions of effect in the dementia and non-dementia groups, thus we presented the results for each group separately. We added S4 Table and S6 Table, and the limitations was revised as follows.

Revised text on page 19, line 369 – 373:

“Fifth, we did not include the results of the non-dementia group in the main table while analyzing the factors associated with polypharmacy. A comprehensive comparison could further elucidate the risk factors for polypharmacy in patients with dementia. However, considering that dementia duration, only applied to patients with dementia, we chose to present the results for patients with and without dementia separately.”

**Minor comments:**

**Comment 1.**

**The manuscript should be reviewed by an English native speaker to improve its general understanding and quality.**

The entire manuscript was revised by an English language professional; the appropriate certificate has been uploaded to Editorial Manager.

**Comment 2.**

**The author should specify the ethic committee and its decision date and decision reference.**

The original text was supplemented with specific information regarding the ethics committees, dates of approval, and clearance numbers (“IRB No.”).

Revised text on page 6, line 105 – 109:

“Ethical clearance for the study was waived by the Institutional Review Board (IRB) of Woosuk University (October 20, 2021, at the beginning of the study; IRB no. ABN01-202305-02-V1). Due to a change in the corresponding author’s affiliation, ethical clearance exemption was again granted by the IRB of Korea National Open University (May 17, 2023; IRB no. ABN01-202305-02-V1).”

**Comment 3.**

**The author used the codes F00, F01, F02, F03, G30 and G31.82, which refer mainly to Alzheimer’s disease. However, these codes do not include cognitive disorder or impairment, which is a major selection bias. Is it possible for the author to include those missing patients? If not, could they provide justification and/or specify in the method section that these patients were not included?**

The definitions in this study were based on KCD codes from previous studies that used health insurance claims data to define patients with dementia. The codes used do not only include Alzheimer’s disease (F00, and G30), but also vascular (F01) and other dementias (F02, F03, and G31.82). We also tried to minimize selection bias by including patients with prescriptions for dementia medications, even if they did not have the aforementioned codes. We made the appropriate revisions in the text and added the definitions for each KCD code as S1 Table. Regarding cognitive disorder or impairment, only mild cognitive impairment could be defined using the KCD code (F06.7). We did not include mild cognitive impairment because no other study included it in their definition of patients with dementia, since mild cognitive impairment has different characteristics from dementia. We considered conducting a sensitivity analysis by including code F06.7 in our definition of dementia patients, but F06.7 was not included in our dataset because it was classified as a sensitive comorbidity and masked by the NHIS. Nevertheless, according to our definition, patients with cognitive disorder or impairment taking dementia medication were included in the dementia group, which we expect would minimize the selection bias.

Revised text on page 6 – 7, line 115 – 121:

“Based on previous definitions in similar studies [33, 34, 38], patients with dementia were identified as those who received at least one treatment with the primary or secondary disease codes F00, F01, F02, F03, G30, G31.82 (Korean Standard Classification of Diseases and Causes of Death-7; KCD-7; S1 Table), or those who were prescribed dementia medication (donepezil, galantamine, rivastigmine, and memantine) at least once in 2019. These criteria included not only patients with Alzheimer’s disease, vascular dementia, and dementia in other diseases, but also patients taking dementia medication without the aforementioned disease code.”

**Comment 4.**

**The author defined disability using the disability grades used in South Korea. Can they provide how these grades are assessed?**

We added a brief description of how disability grades were assessed. Because each type of disability had different rating criteria, we could not fully describe their classification in the text; however, we provided references to the relevant publications, even though these are only available in Korean.

Revised text on page 7, line 136 – 138:

“The disability grade is determined according to the criteria set for each type of disability by the Ministry of Health and Welfare notice when registering a disability [41].”

**Comment 5.**

**Can the author precise the ranking of the quantiles used for the income level? It is only precise in the results section but a phrase such as (“the fifth quantile represents the highest income”) could be added in the method section.**

This may have caused confusion because the ranking of income-level quintiles was not specified in the original Methods section. As suggested, we clarified that the fifth quintile represents the highest income level.

Revised text on page 7, line 131 – 134:

“NHI patient income levels were divided into quintiles based on insurance contribution (the fifth quintile corresponds to the highest contribution), while Medical Aid beneficiaries, representing the lowest income group, were treated as a separate category.”

**Comment 6.**

**In the table 1, the term Institutional care was used whereas “facility service” is used in the method. The authors should stay consistent.**

Based on your note, we revised the sentence as follows.

Revised text on page 8, line 146 – 150:

“The type of long-term care benefits was classified, based on the earliest long-term care benefit records in 2019, as “institutional care” services provided upon admission to a nursing or community living home, “home care” services provided by a visiting caregiver or nurse, and “none” for those who did not receive long-term care benefits.”

**Comment 7.**

**Missing values assessment, analysis and consideration should be provided in the method section.**

Among the 928,561 elderly patients in the dataset as of 2019, residential data was only missing for 13, excluding cases where the date of death was before January 1, 2019. We determined that missing residential data was not a suitable reason for exclusion, thus we imputed the missing information using residential data for 2018 or, if unavailable, 2017. No other variables had missing data.

Revised text on page 7 – 8, line 142-144:

“Most of the study population had no missing values, but a few had missing residential data. For these individuals, we imputed missing values based on the individual’s residential information for the previous year/-s.”

**Comment 8.**

**Can the author precise how the CCI variable was dichotomized? The result section shows 3 categories, but it was not specified why/how these cut-offs were chosen.**

The CCI scores of “0,” “1,” “2,” and “3 or more” were widely used in previous research. However, because dementia has a weight of 2 in the CCI, a different classification was needed. Therefore, 2 was set as the lower cut-off, and subsequent classifications were made based on the CCI distribution of the study population.

Revised text on page 8, line 159 – 161:

“Considering the weight for dementia (i.e., 2) and the CCI distribution of the study population, CCI was categorized as “≤2,” “3–4,” or “≥5.””

**Comment 9.**

**The models used may include redundant variables (CCI and dementia, disability, and dwelling accommodation, …). Have the authors assessed multicollinearity? If so, can they specify it in the method section?**

Based on the variable definitions and distribution, there was no significant concern regarding multicollinearity. Nevertheless, we assessed multicollinearity in all logistic regression models using the generalized variance inflation factor, which was up to 1.059 (adjusted for degrees of freedom), confirming the absence of multicollinearity. We added the appropriate discussion in the text.

Revised text on page 9, line 174 – 176:

“Multicollinearity between covariates in each model was assessed using the generalized variance inflation factor calculated using the car package [44] in R software version 4.3.0 (The R Foundation, Vienna, Austria).”

**Comment 10.**

**The authors should provide in supplementary material comorbidities’ details (overall and groups/sub-groups)**

We included supplementary material on the comorbidities of the study population. This material covers comorbidities used in the CCI (Charlson’s comorbidity index) calculations as well as comorbidities related to dementia. The additional text related to this topic is as follows, and detailed tables can be found in S2, S3 and S5 Tables.

Revised text on page 9, line 177 – 180:

“To better understand polypharmacy patients with dementia, we performed additional analyses on the distribution of some related comorbidities and the associated medication composition. The additional comorbidities were hypertension, depression, and mental health-related diseases other than dementia, based on definition in previous studies [45] and the available data.”

Revised text on page 10, line 206:

“(The detailed distribution of comorbidities included in the CCI is presented in S2 Table.)”

Revised text on page 11 – 12, line 225 – 228:

“The detailed distribution of comorbidities included in the CCI is shown in S3 Table. The prevalence of congestive heart failure, chronic pulmonary disease, mild liver disease, diabetes with chronic complications, and renal disease was significantly higher in patients with excessive polypharmacy than in those without.”

Revised text on page 14, line 235 – 237:

“Based on the CCI, higher comorbidity rates were observed in patients with polypharmacy and excessive polypharmacy than in those without (S5 Table).”

Revised text on page 15, line 260 – 263:

“Regarding the related comorbidities, patients with dementia had a relatively higher prevalence of depression and mental health-related diseases than patients without dementia, and even among patients with dementia, the patients with polypharmacy and excessive polypharmacy had a relatively higher proportion of these comorbidities (S2, S3, and S5 Tables).”

**Comment 11.**

**In the discussion, the authors assumed that the difference of polypharmacy in dementia patients may be because of the prescription of BPSD controlling drugs. As the author have access to prescriptions records, it will strengthen authors’ conclusion if drug classes comparison was provided.**

Thank you for your helpful comments. We analyzed the proportion of medications associated with BPSD in polypharmacy and found a higher proportion of antipsychotics and antidepressants among dementia patients compared to non-dementia patients. We added this as supplementary material (S8 Table) and updated the discussion accordingly. In order to perfectly fit the discussion, analyzing data by dementia duration might have been ideal, but we considered it beyond the scope of this study. Therefore, we supplemented our findings with an analysis of dementia patients and non-dementia patients.

Revised text on page 16 – 17, line 292 – 297:

“Second, the likelihood of polypharmacy and excessive polypharmacy increased as the duration of dementia increased even after controlling for age and CCI, which was not reported in previous studies. This suggests that more medications were prescribed for treating the behavioral and psychological symptoms of dementia the duration of dementia increased. This possibility is supported by the higher proportion of antipsychotics and antidepressants in the medication composition of polypharmacy in patients with dementia (S7 Table).”

**Comment 12.**

**In the discussion, the authors discussed the issue of income when comparing Medical Aid vs NHI population. However, the analysis showed an OR < 1 for NIH 1 to 3 compared when compared to NIH 5. Could this author add a section to discuss this result as well?**

In Korea, Medicare Aid patients pay low out-of-pocket costs for healthcare utilization and prescribed medications compared to NHI patients. We thought this might have influenced our results, so we supplemented the discussion as follows.

Revised text on page 24, line 437 – 450:

“Third, the likelihood of polypharmacy and excessive polypharmacy was especially high among Medical Aid beneficiaries, the lowest-income group, consistent with previous findings on elderly patients in South Korea [49, 56]. While previous studies compared NHI with Medical Aid beneficiaries, we further divided the NHI population into income quintiles but found no difference in the likelihood of polypharmacy and excessive polypharmacy between income quintiles in patients with dementia. The significantly higher likelihood of polypharmacy and excessive polypharmacy in Medical Aid compared to NHI beneficiaries may be due to differences in benefit systems or health status rather than solely income disparities. Because Medical Aid beneficiaries have low out-of-pocket healthcare costs, they may be able to use medical care and receive prescriptions for mediations more easily, thereby increasing the possibility of polypharmacy.”

**Comment 13.**

**This author should give practical implication of their findings.**

We updated our thinking on this as follows.

Revised text on page 18, line 325 – 331:

“Fourth, both patients with and without dementia had a high likelihood of polypharmacy when receiving long-term care benefits. Compared to patients not receiving long-term care benefits, the likelihood of polypharmacy and excessive polypharmacy was significantly higher in patients receiving home care, and the likelihood of polypharmacy was significantly higher in patients receiving institutional care. Raising awareness of polypharmacy by educating long-term care providers about the potential risks could contribute to quickly recognizing and responding to the risks that may arise due to polypharmacy.”

**Response to the Comments from Reviewer 2**

**Comment 1.**

**Clarity in Introduction: The introduction could benefit from a clearer articulation of the research gap. While it mentions the scarcity of studies in this area, a more explicit statement of what specifically this study adds would be helpful.**

Thank you for helpful comments. We revised the introduction to make it more explicit about the research gap and what this study adds.

Revised text on page 5, line 72 – 89:

“Consequently, the management of patients with dementia has become a crucial clinical service [26-28]. In South Korea, one of the fastest aging countries, 10.3% (924,870 people) of the population aged≥65 years suffered from dementia in 2022, which is 1.7-times higher than that in 2012. The prevalence of dementia in South Korea was predicted to increase by another 1.6-fold by 2032 [29, 30]. Consequently, dementia has gained national research interest in South Korea, especially regarding its pharmacological treatment [31-36]. Nevertheless, no study has addressed the characteristics of polypharmacy in patients with dementia. The study of polypharmacy in patients with dementia could provide valuable information not only for clinical practitioners and policy makers in Korea, where the population is aging rapidly, but also in other countries with slower population aging rates. In addition, a recent systematic literature review found that the prevalence of polypharmacy in patients with dementia varied considerably between regions [23]. This may reflect the influence of region-specific healthcare systems and the different settings of each study. The results of the study using representative data from Korea are expected to add additional evidence on polypharmacy in dementia patients, especially to the lack of evidence in Asian countries. To bridging this gap in knowledge, we aimed to determine the status and associated factors of polypharmacy among outpatients with dementia in 2019, using data sourced from the National Health Insurance Service (NHIS)-Senior cohort database. To elucidate the characteristics, prevalence, and risk factors of polypharmacy in dementia, we compared the data between patients with and without dementia.”

**Comment 2.**

**Broader Contextualization: The discussion could explore how these findings relate to or differ from studies in other countries, especially those with different healthcare systems or demographic profiles.**

In response to your comment, we added the following to the discussion.

Revised text on page 18, line 332 – 339:

“Finally, our comparison of patients with and without dementia elucidated the characteristics of polypharmacy in this cohort along with important factors that affect its prevalence in patients with dementia. Since age, disability, Medical Aid beneficiaries, and CCI had greater effects on polypharmacy in patients without dementia (S6 Table), dementia itself may be a risk factor for polypharmacy. The higher risk for polypharmacy in patients with dementia compared to those without is consistent with findings from the United Kingdom (patients aged ≥65 years registered at general practices) [8] and United States (outpatients aged ≥65 years) [21], though the cohorts in these studies had different demographic profiles and healthcare systems.”

**Comment 3.**

**Addressing Limitations: While some limitations are implicit (such as the study's focus on South Korea), it would be beneficial for the authors to explicitly state these limitations and discuss how they might affect the generalizability of the findings.**

We supplemented the discussion by reflecting your comments. This part is also connected to Comment 4 below, and we supplemented them together. Please refer to the response to Comment 4 and the revised text below.

**Comment 4.**

**Potential Biases: Any potential biases in the data source or methodology should be acknowledged and discussed, such as selection bias in the cohort or limitations in the diagnostic codes used.**

We described in more detail the characteristics of the data we used and presented potential biases as limitations. In addition, we also provided further explanation of the generalizability limitations of our study in relation to Comment 3. The added text is as follows.

Revised text on page 19, line 363 – 369:

“Fourth, the data source used in this study was sampled to represent 60–80-year-old population in 2008. Although a new sample of the population turning 60-year-old was added every year until 2019, our cohort may not strictly represent the elderly in Korea as of 2019. However, to include elderly of over 80 years and analyze the most recent situation, we analyzed data from 2019. Even though we attempted to include the maximum possible age within the data, the oldest individual in 2019 was 91; therefore, our study findings do not represent elderly individuals aged≥92 years. This along with the fact that we only considered Korean patients, limits the generalization of our results.”

**Comment 5.**

**Implications for Future Research: The article could offer more detailed suggestions for future research, such as exploring interventions to reduce polypharmacy risks or examining the impact of polypharmacy on specific outcomes in dementia patients.**

We supplemented the discussion regarding future research as follows.

Revised text on page 20, line 374 – 378:

“Despite these limitations, we used the same framework for study polypharmacy in patients with and without dementia. Our comparison between patients with and without dementia suggested that polypharmacy should be approached differently between these patient populations, barring further qualitative research on the individual appropriateness of polypharmacy. Further research is also needed on the long-term consequences of polypharmacy in dementia patients.”

**Comment 6.**

**Abstract Structure: The abstract could be structured more clearly with distinct sections for background, methods, results, and conclusions to enhance readability.**

Thank you for your kind comments. We structured the abstract using the appropriate titles.

Revised Abstract on page 2 – 3, line 17 – 41:

The revised abstract is not cited here.

**Comment 7.**

**Consistency in Terminology: Ensure consistency in the use of terms and definitions throughout the paper.**

In our revision, we made concerted efforts to promote terminological consistency throughout the manuscript. We also revised various definitions for clarity and consistency.

**Comment 8.**

**References: Check for the latest references, especially if there have been significant developments in the field since the original literature review was conducted.**

Thank you for your helpful comments. Following our initial literature review during the paper writing process, we conducted an additional search of newly published literature. In the course of this process, we discovered a systematic review published in 2023 that included papers on polypharmacy in dementia patients. Through this study, we confirmed that there remains a gap in research regarding polypharmacy in dementia patients. We incorporated this information to enhance the introduction. The supplemented text is the same as the text below Comment 1, so we did not add it separately.
